# ﻿Revision of the xyleborine ambrosia beetle genus *Microperus* Wood, 1980 (Curculionidae, Scolytinae, Xyleborini) of Thailand with four new species and four newly recorded species

**DOI:** 10.3897/zookeys.1074.76235

**Published:** 2021-12-03

**Authors:** Wisut Sittichaya, Sarah M. Smith, Roger A. Beaver, Narit Thaochan

**Affiliations:** 1 Agricultural Innovation and Management Division, Faculty of Natural Resources, Prince of Songkla University, Songkhla, 90110, Thailand Prince of Songkla University Songkhla Thailand; 2 Department of Entomology, Michigan State University, East Lansing, MI 48824, USA Michigan State University East Lansing United States of America; 3 161/2 Mu 5, Soi Wat Pranon, T. Donkaew, A. Maerim, Chiangmai 50180, Thailand unaffiliated Chiangmai Thailand

**Keywords:** Key, *Microperus*, new combinations, new records, new species, new synonymy, Oriental region, Thailand

## Abstract

*Microperus* Wood, 1980 ambrosia beetles in Thailand are reviewed. Four species, *M.bidentatus***sp. nov**., *M.bucolicus***sp. nov**., *M.globodeclivis***sp. nov.**, and *M.serratus***sp. nov.** are described. Four new combinations are given: *Microperusarmaticeps* (Schedl, 1942) **comb. nov.**, *Microperusexsculptus* (Eggers, 1927) **comb. nov.**, *Microperuspedellus* (Schedl, 1969) **comb. nov.**, and *Microperusspicatulus* (Browne, 1986) **comb. nov.**, **stat. res.**, all from *Xyleborus*. Two new synonyms are proposed: *Microperuscruralis* (Schedl, 1975) (= *Xyleborusmyllus* Browne, 1986 **syn. nov.**), *Microperusexsculptus* (Eggers, 1927) (= *Xyleborusdentipennis* Browne, 1983 **syn. nov.**). Four species are reported from Thailand for the first time: *Microperuschrysophylli* (Eggers, 1930), *Microperusexsculptus*, *Microperusnanus* (Browne, 1949) and *Microperusquercicola* (Eggers, 1926). With the inclusion of the *Microperus* species described and recorded herein, the diversity of *Microperus* is increased to 35 species, of which 18 are recorded in Thailand. An updated key to the *Microperus* of the Indochinese Peninsula and China is provided. The taxonomy, diagnostic characters, and distribution of species are briefly discussed.

## ﻿Introduction

The xyleborine ambrosia beetle genus *Microperus* was first erected by Wood (1980) for members of the *Xyleborustheae* Eggers, 1940 species group, but the genus name was initially proposed in an unpublished manuscript by F. G. Browne (Wood 1980). Wood diagnosed *Microperus* species as slender, at least 2× as long as wide, posterior face of the antennal club with at least one suture visible and apical margin of corneous area never costate, scutellum invisible and strial punctures usually seriate. Later Wood (1986) synonymized *Microperus* with the similar genus *Coptodryas* Hopkins, 1915 without comment. *Microperus* was resurrected and separated from *Coptodryas* Hopkins, 1915 as a result of a cladistic review of xyleborine taxonomic characters (Hulcr et al. 2007). The genera are distinguished by multiple differences in the antennal characters, and body shape. Subsequent molecular phylogenetic studies including both *Coptodryas* and *Microperus* have confirmed that the genera are closely related (Cognato et al. 2011; Mandelshtam et al. 2019). However, Hulcr et al.’s (2007) diagnosis and description of both *Coptodryas* and *Microperus* were based on a limited taxon sampling of five species per genus and as a result, did not reflect the full morphological variation exhibited by species within these genera. Smith et al. (2020) recently expanded Hulcr et al.’s (2007) work in a revision of the fauna of Indochina and China and provided revised descriptions and diagnoses. Despite these recent advances, additional revisionary work is required to identify other *Microperus* species still erroneously placed in *Coptodryas*.

*Microperus* currently contains 27 species distributed in Asia from Far East Russia, Korea and Japan, west to Sri Lanka and southeast to Papua New Guinea and Australia. Eighteen species are distributed in Indochina and China, and Thailand has the greatest diversity (Smith et al. 2020). The first *Microperus* species recorded in Thailand was *M.pometianus* (Schedl 1939), reported as *Xyleboruspometianus* (Nunberg and Chûjô 1961). Several researchers have subsequently recorded additional species from Thailand (Browne 1980; Beaver 1999; Hutacharern et al. 2007; Sittichaya et al. 2012). The first synoptic list of Thai *Microperus* included nine species (Beaver et al. 2014). Smith et al. (2020) added three additional species, increasing the *Microperus* diversity in Thailand to 12. In this paper, we describe four new species of the genus from Thailand, transfer four species from *Coptodryas* and *Cyclorhipidion* Hagedorn, 1912 to *Microperus* and place two species into synonymy, increasing the total number of *Microperus* species to 35. We also provide a key to the species of *Microperus* in the Indochinese peninsula and China updated from that in Smith et al. (2020).

## ﻿Materials and methods

**Figure 1.**
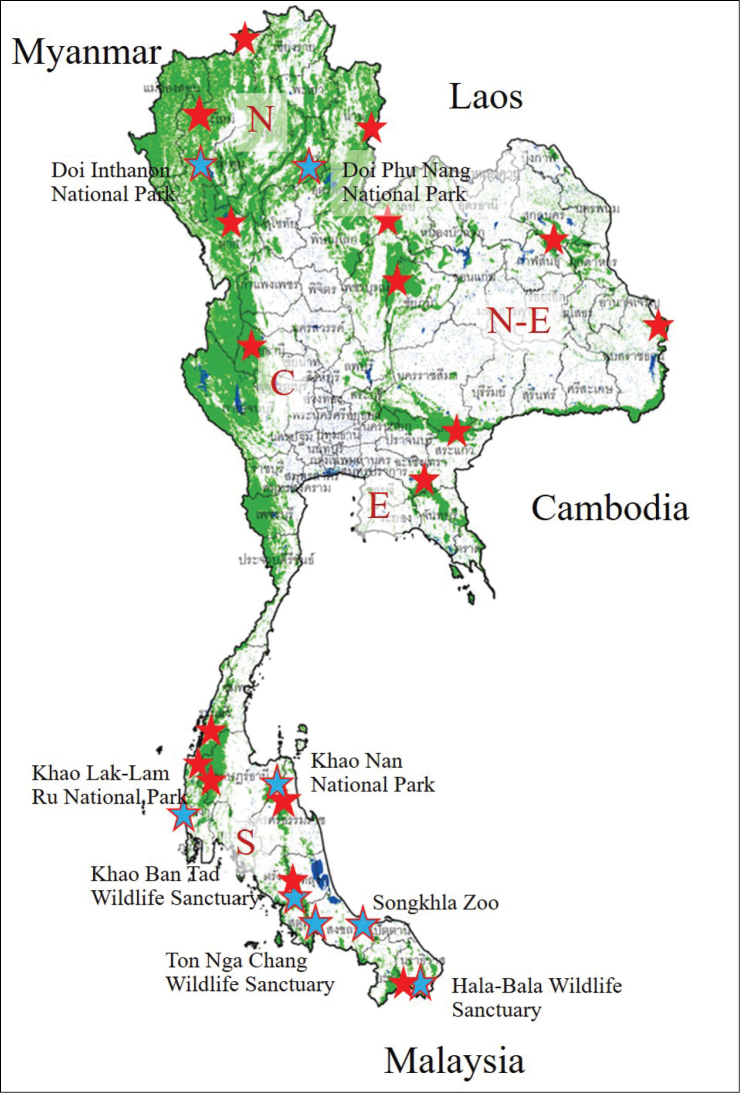
Insect survey localities in Thailand, red stars indicate surveyed areas, blue stars indicate locations where new species and newly recorded *Microperus* species were captured. Source: the map was modified from a Royal Forest Department of Thailand map.

Specimens were collected from 27 study sites in 24 conservation areas across all regions of Thailand. Southern Thailand was sampled between 2014–2015 and the North, Northeast and East were sampled between 2019–2020 (Fig. 1). In Southern Thailand 20 ethanol-baited flight intercept traps were deployed in each study site, whereas five ethanol-baited cross-vane panel traps were used at each site in the other parts of the country (N, NE, E). *Microperus* specimens were collected from Hala-Bala Wildlife Sanctuary, Narathiwat Province; Ton Nga Chang Wildlife Sanctuary, Songkhla Province; Khao Ban Tad Wildlife Sanctuary, Trang province; Khao Nan National Park, Nakhon Sri Thammarat Province, Songkhla Zoo: Songkhla Province, Khao Lak-Lam Ru National Park, Phang Nga Province; Doi Inthanon National Park, Chiang Mai Province and Doi Phu Nang National Park, Phayao Province.

Photographs were taken with a Canon 6D digital Camera with a Canon MP-E 65 mm Macro Photo Lens (Canon, Tokyo, Japan) and StackShot-Macrorail (Cognisys Inc, Michigan, USA). The photos were then combined with Helicon Focus 6.8.0. (Helicon Soft, Ukraine); all photos were improved with Adobe Photoshop CS6 (Adobe Systems, California, USA). The antennal and pronotum types and characters follow those proposed by Hulcr et al. (2007) and subsequently elaborated upon by Smith et al. (2020). Length was measured from pronotum apex to the apex of the declivity and width was measured at the widest part of specimen.

### ﻿Abbreviations used for entomological collections

**MSUC** Albert J. Cook Arthropod Research Collection, Michigan State University, East Lansing, USA;

**NHML**Natural History Museum, London, U.K.;

**NHMW**Museum of Natural History of Vienna, Austria;

**NMNH**National Museum of Natural History, Washington D.C., USA;

**RABC** Roger A. Beaver collection, Chiang Mai, Thailand;

**THNHM** Natural History Museum of the National Science Museum, Thailand;

**WSTC** Private collection of Wisut Sittichaya, Songkhla, Thailand.

## ﻿Taxonomic treatment

### ﻿ Microperus

Taxon classificationAnimaliaColeopteraCurculionidae

Wood, 1980

B53F4E8A-A98F-5C52-BB8E-062533B958A6

Microperus Wood, 1980: 94.

#### Type species.

*Xyleborustheae* Eggers, 1940 (= *Xyleborusmyristicae* Schedl, 1939); original designation.

#### Diagnosis.

This genus is distinguished by the following combination of characters: small to minute size, 1.2–3.1 mm long and cylindrical and elongate form, 1.9–3.2× as long as wide; antennal club truncate or flattened, segment 1 costate, segment 2 or segments 2 and 3 visible on anterior side small and appearing soft, segment 2 or segments 2 and 3 visible on posterior side (types 2, 3, 4 of Hulcr et al. 2007 and Smith et al. 2020); elytral base sinuate, rarely transverse, with a dense tuft of setae present along elytral base associated with an elytral mycangium; scutellum minute or not apparent; strial and interstrial punctures arranged in parallel rows, striae punctate, prominent; pronotum from lateral view taller than basic (type 2 of Hulcr et al. 2007) or with pronotal disc longer than anterior slope (type 7); pronotum from dorsal view basic and parallel-sided (type 2), or subquadrate (type 3), and anterior margin of pronotum without a row of serrations.

#### Similar genera.

*Coptodryas*, *Xyleborinus*

##### New species

### ﻿ Microperus bidentatus

Taxon classificationAnimaliaColeopteraCurculionidae

Sittichaya, Smith & Beaver sp. nov.

06A3CB04-3D76-5F7D-9A70-C47BA4B2E136

http://zoobank.org/2F84550F-FF90-4485-955E-C3B5DE6783EE

Figure 2

#### Type material.

***Holotype***, female, Thailand, Trang Province, Khao Banthat Wildlife Sanctuary, 7°24'54.6"N, 99°49'47.5"E, tropical rainforest, ethanol baited trap, 01.xii.2013, W. Sittichaya, (NHMW). ***Paratype***, female, Nakhon Sri Thammarat Province, Khao Nan National Park, 8°48'31.1"N, 99°33'28.4"E, tropical rainforest, ethanol baited trap, 01.xii.2013, (W. Sittichaya), (1 WSTC).

**Figure 2.**
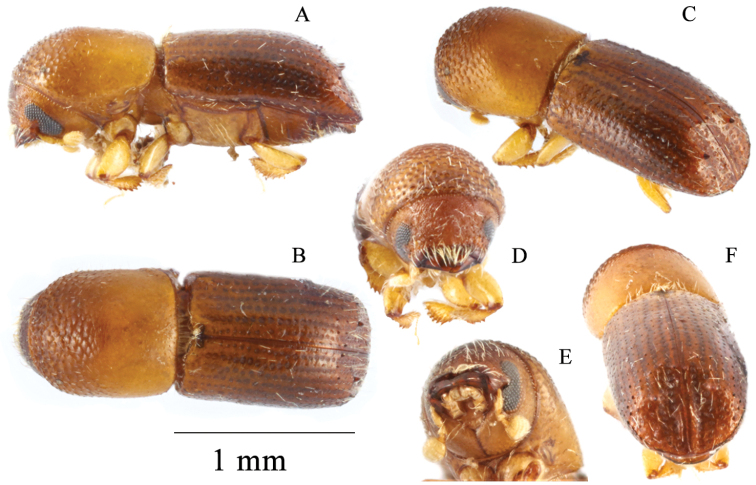
*Microperusbidentatus* sp. nov. holotype **A** lateral view, **B** dorsal view, **C** posterolateral view, **D** front, **E** head ventral view, **F** declivity.

#### Similar species.

*M.recidens*.

#### Diagnosis.

1.68–1.76 mm (mean = 1.72 mm; n = 2) long, 2.80–2.89 (mean 2.85× as long as wide). This species is distinguished by its cylindrical appearance, elytral disc shining and convex, declivity steep, declivital interstriae 1 and 2 flat, slightly impressed, declivital interstriae 3–6 convex on basal half, with a pair of prominent tubercles on interstriae 3, apical half flattened. This species differs from *M.recidens* by the elytral disc more flattened, declivital summit less steep, declivital face more flattened and broader, and declivity armed with a pair of prominent tubercles on interstriae 3 (those much smaller in *M.recidens*).

#### Description (female).

Body greenish brown to brown. ***Head***: epistoma entire, transverse, with a row of hair-like setae. Frons weakly convex shagreened, covered with sparse vestiture, punctate, punctures shallow with a short erect hair-like setae. Eye shallowly emarginate just above antennal insertion, upper portion slightly smaller than lower part. Submentum large, distinctly triangular, moderately impressed. Antennal scape short and thick, subequal in length with club. Pedicel slightly broader than scape, shorter than funicle. Funicle 4-segmented, segment 1 shorter than pedicel. Club longer than wide (5:4), type 3, segment 1 corneous, feebly convex on anterior face, occupying basal 1/3, covering 2/3 of posterior face; segment 2 short, soft; segment 2 present on posterior face, soft. ***Pronotum***: 1.20× as long as wide, elongate and subquadrate, type 7 in dorsal view, lateral sides parallel to anterior 3/4, rounded anteriorly; anterior margin without serrations; base weakly concave, posterior angles rounded. In lateral view disc slightly longer than anterior slope, type 7, summit at apical 2/5, anterior slope densely asperate; disc slightly convex, subshiny, impunctate; lateral margins obliquely costate. ***Elytra***: 1.55× as long as wide, 1.33× as long as pronotum. Scutellum minute, convex, slightly raised above elytral surface. Elytral mycangium indicated by a disperse median setal tuft along elytral base. Base sinuate, edge carinate, humeral angles rounded, parallel-sided in basal 7/8, then narrowly rounded to apex. Disc shiny, moderately convex, striae not impressed, with large shallow punctures, setose, setae very short, semi-erect, hair-like; interstriae flat, impunctate, setose, setae long erect, hair-like. Declivity abruptly commencing, declivital face on striae and interstriae 1 and 2 shallowly impressed, upper portion on 3–6 convex with a pair of prominent tubercles on interstriae 3, lower portion flattened; declivital striae 1 and 2 slightly impressed, punctate, punctures round, prominent, moderately impressed, striae 3–6 flat, punctate, punctures small; interstriae flat with a row of long erect hair-like setae. Posterolateral margin carinate to interstriae 7. ***Legs***: procoxae contiguous; prosternal coxal piece short, inconspicuous. Protibiae slender, broadest at the middle; posterior face smooth; margin armed with six large socketed denticles. Meso- and metatibiae rounded, armed with seven large socketed denticles.

#### Etymology.

L. *bi* = two, *dentatus* = toothed, the name refers to the two prominent denticles on each declivital interstriae 3. An adjective.

#### Distribution.

Southern Thailand (Trang Province, Nakhon Sri Thammarat Province).

#### Host plants.

Unknown.

### ﻿ Microperus bucolicus

Taxon classificationAnimaliaColeopteraCurculionidae

Sittichaya, Smith & Beaver sp. nov.

8EA3E55D-BF1D-582A-A59C-92378559C0CA

http://zoobank.org/8AD72B58-7FF5-4CD0-97C1-EC006009329A

Figure 3

#### Type material.

***Holotype***, female, Thailand: Nakhon Sri Thammarat Province, 8°21'40.8"N, 99°39'17.3"E, durian orchard, ethanol baited trap, 1.vi.2011, W. Sittichaya, (MSUC).

#### Diagnosis.

1.6 mm (n = 1), 2.67× as long as wide. This species is distinguished by the elytral disc flat with short, steep declivity, declivital posterolateral margin costate and denticulate, declivity with sparse minor denticles, less abundant than strial punctures, and denticles uniform in size.

**Figure 3.**
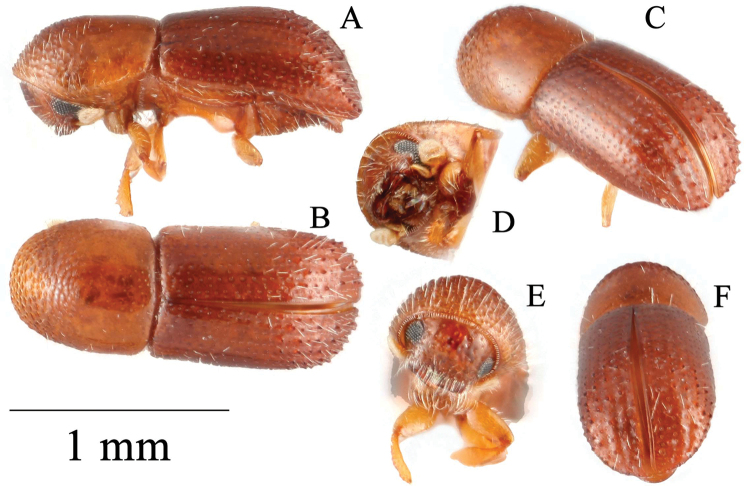
*Microperusbucolicus* sp. nov. holotype **A** lateral view **B** dorsal view **C** posterolateral view **D** head ventral view **E** front **F** declivity.

#### Similar species.

*M.alpha*.

#### Description (female).

Head, pronotum and elytra light brown, antennae and legs yellow brown. ***Head***: epistoma entire, transverse, with a row of hair-like setae. Frons weakly convex to upper level of eyes, subshiny, punctate; punctures large, shallow, sparse, each bearing an erect hair-like seta. Eyes moderately emarginate just above antennal insertion, upper part smaller than lower part. Submentum large, distinctly triangular, slightly impressed. Antennal scape short and thick, shorter than club. Pedicel as wide as scape, shorter than funicle. Funicle 4-segmented, segment 1 shorter than pedicel. Club longer than wide, flattened, type 3; segment 1 corneous, transverse on anterior face, occupying basal 1/4 of club; segment 2 narrow, soft; segments 1 and 2 present on posterior face. ***Pronotum***: 1.09× as long as wide. In dorsal view basic and parallel-sided (type 2), sides parallel in basal 2/3, rounded anteriorly; anterior margin without serrations. Base weakly bisinuate, posterior angles obliquely rounded. In lateral view elongate with disc much longer than anterior slope, type 8, summit low, at apical 2/5. Anterior slope with densely spaced, broad asperities, becoming lower and more strongly transverse towards summit. Disc shagreened, alutaceous, finely punctate, glabrous, some moderately long hair-like setae at margins. Lateral margins obliquely costate. ***Elytra***: 1.53× as long as wide, 1.4× as long as pronotum. Scutellum minute, convex, slightly raised above elytral surface. Elytral mycangium indicated by a disperse median setal tuft along elytral base. Base transverse, edge oblique, humeral angles rounded, parallel-sided in basal 4/5, then narrowly rounded to apex. Disc flat, shiny, striae clearly impressed, with large deep punctures separated by 2–3 diameters of a puncture, glabrous; interstriae flat, impunctate, setose, setae short, sparse, erect hair-like. Declivity occupying 1/3 of elytral length, steeply rounded, its margins denticulate, weakly shagreened, subshiny; striae flat, punctate, each puncture bearing a seta as long as a puncture; interstriae regularly denticulate along their lengths, denticles uniformly sized, each bearing a long erect hair-like seta. Posterolateral margin costate, denticulate to interstriae 7. ***Legs***: procoxae contiguous; prosternal coxal piece tall, pointed. Protibiae slender, broadest at the middle; posterior face smooth; margin armed with at least six large socketed denticles (broken).

#### Etymology.

L. *bucolicus* = rural, rustic. In reference to the agrarian habitat in which the species was collected. An adjective.

#### Distribution.

Southern Thailand (Nakhon Sri Thammarat Province).

#### Host plants.

Unknown.

### ﻿ Microperus globodeclivis

Taxon classificationAnimaliaColeopteraCurculionidae

Sittichaya, Smith & Beaver sp. nov.

29B9E92E-9228-5A29-ADDD-14059867ED9F

http://zoobank.org/CAF6E1EF-5095-4B32-A17A-3C0ABCAAA609

Figure 4

#### Type material.

***Holotype***, female, Thailand: Payao Province, Doi Phu Nang National Park, 18°51'57.7"N, 100°10'51.0"E, dry deciduous dipterocarp forest, ethanol baited trap, 01.i.2019, W. Sittichaya (NHMW).

#### Diagnosis.

1.6 mm long (n = 1), 2.66× as long as wide. This species is distinguished by its cylindrical appearance, convex elytral disc and declivity, elytral apex globose and posterolateral margin broadly rounded without a carina. This species is differentiated from the morphologically similar *M.pedellus* (Schedl, 1969) by the following combination of characters (*M.globodeclivis* given first): less elongate form, 2.66× as long as wide vs. 2.8× as long as wide), stouter pronotum 1.08× as long as wide vs. 1.17× as long as wide, elytra 1.5× as long as pronotum vs. 1.4× as long as pronotum, and elytral base sinuate vs. broadly V-shaped.

**Figure 4.**
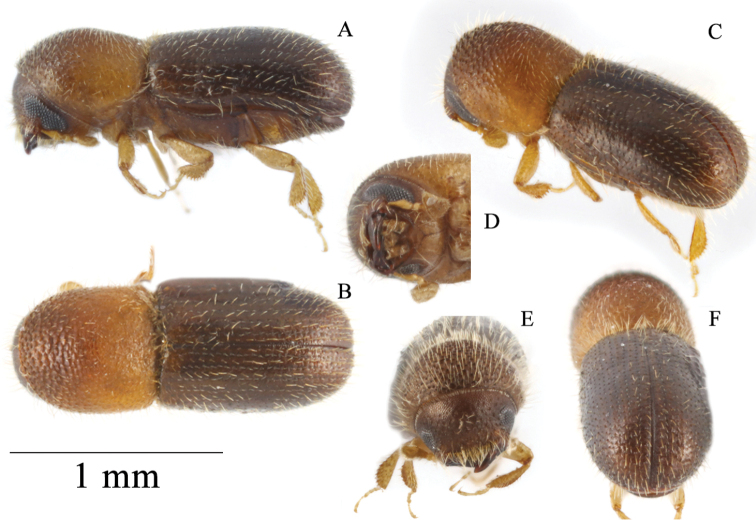
*Microperusglobodeclivis* sp. nov. holotype **A** lateral view **B** dorsal view **C** posterolateral view **D** head ventral view **E** front **F** declivity.

#### Similar species.

*M.pedellus*.

#### Description (female).

Appearing bicolored, moderately setose: head, anterior slope of pronotum and elytra dark brown, remainder of pronotum, antennae, and legs light brown. ***Head***: epistoma entire, transverse, with a row of dense hair-like setae. Frons weakly convex to upper level of eyes, shagreened lower part densely covered with long erect hair-like setae, punctate, punctures shallow and bearing a long, erect hair-like seta. Eye shallowly emarginated just above antennal insertion, upper and lower parts subequal. Submentum medium in size, distinctly triangular, shallowly impressed. Antennal scape short and thick, as long as club. Pedicel as wide as scape, shorter than funicle. Funicle 4-segmented, segment 1 shorter than pedicel. Club circular, broader than tall; type 3; segment 1 corneous covering about half of posterior face, anterior face costate concave, narrow; segment 2 narrowly corneous on anterior face, posterior face fully visible, segment 3 soft, visible on posterior face. ***Pronotum***: 1.08× as long as wide. In dorsal view basic type 2; lateral side parallel to anterior two thirds; anterior margin round without medial serrations. Base weakly bisinuate, posterior angles acutely rounded. In lateral view type 7, disc slightly longer than anterior slope; disc convex, summit at apical 2/5; anterior slope densely covered with asperities; disc shagreened, impunctate; pronotum sparsely covered with moderately long hair-like setae, lateral margins obliquely costate. ***Elytra***: 1.62× as long as wide, 1.5× as long as pronotum. Scutellum minute, convex, slightly raised above elytral surface. Elytral mycangium indicated by a disperse median setal tuft along elytral base. Base shallowly sinuate, edge oblique, humeral angles rounded. Lateral margins parallel beyond basal half, apex broadly attenuated at apical 4/5, apex broadly rounded. Declivity gradually commencing, broadly convex without posterolateral carina; elytra setose, elytral disc shinning, flat near scutellum broadly convex beyond, striae punctate, parallel laterally, puncture small, round moderately deep, punctures with shorter hair-like setae, interstriae flat, impunctate bearing a row of long erect hair-like setae, setae on disc and declivity equal in length. ***Legs***: procoxae contiguous, prosternal coxal piece short, inconspicuously covered with a tuft of long hair-like setae. Protibiae slender, broadest at the middle; posterior face smooth; margin armed with six small socketed denticles. Meso- and metatibiae inflated; outer margin evenly rounded with seven and six small socketed denticles, respectively.

#### Etymology.

L. *globus* = globe, round; *declivis* = downhill sloping (declivity). In reference to its round declivity which lacks a posterolateral carina. An adjective.

#### Distribution.

Northern Thailand (Payao Province).

#### Host plants.

Unknown.

### ﻿ Microperus serratus

Taxon classificationAnimaliaColeopteraCurculionidae

Sittichaya, Smith & Beaver sp. nov.

D2C82B70-EC6D-5BAF-82A6-E5DCF691B194

http://zoobank.org/B8E6B1EB-89F9-466C-88B9-F4785B2FF2CE

Figure 5

#### Type material.

***Holotype***, female, Thailand: Songkhla Province, Ton Nga Chang Wildlife Sanctuary, 6°59'32.1"N, 100°08'57.8"E, tropical rainforest, ethanol baited trap, 01.iii.2015, (W. Sittichaya) (NHMW). ***Paratypes***, female, same data as holotype, 01.iv.2014 (1) (THNHM), 01.vii.2014 (1) (WSTC); Songkhla Province, Songkhla Zoo, 7°08'53.7"N, 100°36'29.4"E, secondary tropical rainforest, ethanol baited trap, 01.iii.2019, (1) (WSTC); Narathiwat Province, Hala-Bala Wildlife Sanctuary, 5°47'44"N, 101°50'07"E, lowland tropical rainforest, ethanol baited trap, 01.ii.2015 (1) (RABC) (1) (MSUC). Malaysia: Sabah, Sipitang, Mendolong, A1L, 5.iv.1988, S. Adebratt (1) (RABC); as previous except: 6.iv.1988 (1) (RABC).

#### Similar species.

*M.nugax*, *M.sagmatus*, *M.undulatus* (Sampson, 1919).

#### Diagnosis.

1.66–1.72 mm long (mean = 1.70 mm; n = 6) 2.59–2.69× as long as wide (mean 2.64× as long as wide). This species is distinguished by elytral disc convex, posterior half of elytra striae impressed with large round punctures, impression increased posteriorly, interstriae costate with strong interstrial spines posterior to declivital summit, declivity steep, shallowly impressed on the middle to interstriae 2, interstriae 3 with small but prominent tubercles.

**Figure 5.**
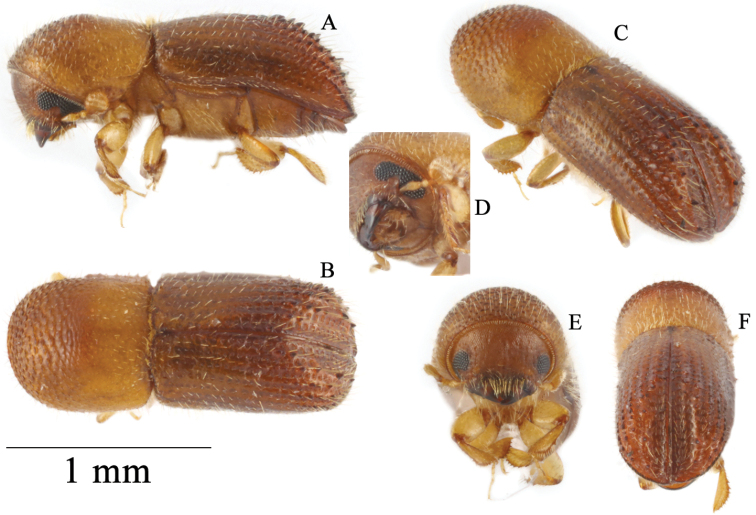
*Microperusserratus* sp. nov. holotype **A** lateral view **B** dorsal view **C** posterolateral view **D** head ventral view **E** front **F** declivity.

This species is related to *M.undulatus* and similar species, but elytral disc is convex, lacking a transverse or saddle-like impression.

#### Description.

***Head***: epistoma entire, transverse, with a row of hair-like setae. Frons subshiny, finely punctate, lower portion flat sparsely covered with very fine hair-like setae, upper level of eyes slightly convex; sparsely setose, setae fine. Eye shallowly emarginate just above antennal insertion, upper part smaller than lower part. Submentum large, distinctly triangular, slightly impressed. Antennal scape short and thick, as long as club. Pedicel as wide as scape, shorter than funicle. Funicle 4-segmented, segment 1 shorter than pedicel. Club longer than wide, type 3, anterior face segment 1 corneous, concave, segment 2 narrowly visible, posterior face segment 1 covering basal 2/3, segment 2 visible, appearing soft. ***Pronotum***: 1.05× as long as wide. In dorsal view subquadrate and parallel-sided, type 3, sides parallel in basal 2/3, weakly rounded anteriorly with prominent anterolateral corners; anterior margin without a row of serrations. Base weakly bisinuate, posterior angles acutely rounded, almost subquadrate. In lateral view tall, type 2, disc flat, summit at midpoint. Anterior slope with densely spaced, broad asperities, becoming lower and more strongly transverse towards summit. Disc shagreened, alutaceous, impunctate, lateral margins obliquely costate. ***Elytra***: 1.53× as long as wide, 1.53× as long as pronotum, Scutellum minute, convex, slightly raised above elytral surface. Elytral mycangium indicated by a disperse median setal tuft along elytral base. Base shallowly sinuate, edge oblique, humeral angles rounded, parallel-sided in basal 4/5, then narrowly rounded to apex. Disc shiny, basal half convex, striae and interstriae flat, posterior half rugose with deeply impressed striae and costate interstriae bearing strong incurved spines, becoming more prominent toward declivital summit which bears the largest incurved spine. Declivity steep, declivital face dull, striae and interstriae 1 and 2 shallowly impressed, striae 1 and 2 with prominent round punctures, interstriae 3 with two small acute denticles. Striae with short erect hair-like setae and interstriae with long setae. Posterolateral margin acutely carinate to interstriae 7. ***Legs***: procoxae contiguous; prosternal coxal piece tall, conical. Protibiae obliquely triangular, broadest at apical 1/3; posterior face smooth; apical 1/3 of outer margin with six moderate socketed denticles. Meso- and metatibiae flattened; outer margin evenly rounded with eight moderate to large socketed denticles.

#### Etymology.

L. *serratus* = toothed like a saw. In reference to the serrate margin of the elytral declivity. An adjective.

#### Distribution.

Thailand (Narathiwat Province, Songkhla Province), East Malaysia (Sabah).

#### Host plants.

Unknown.

##### New combinations and new synonymy

### ﻿ Microperus armaticeps

Taxon classificationAnimaliaColeopteraCurculionidae

(Schedl, 1942) comb. nov.

B18D7D49-5638-5D7A-99A8-96634586A556

Xyleborus armaticeps Schedl, 1942: 198.
Cyclorhipidion armaticeps (Schedl, 1942): Wood and Bright 1992: 698.

#### Remarks.

A dorsal habitus image of the lectotype (NMHW) was examined by all authors and the species is transferred from *Cyclorhipidion* to *Microperus* because of the following characters: dense tuft of setae present along elytral base associated with an elytral mycangium; elytral bases broadly V-shaped, costate; scutellum minute, convex and slightly raised above elytra; pronotum from dorsal view basic and parallel sided (type 2), from lateral view disc longer than anterior slope (type 7), and pronotal base weakly sinuate.

This species is a probable synonym of *M.exsculptus* (Eggers, 1927) but has larger denticles along the declivital margin. Additional investigations are needed to assess intraspecific variation and clarify species limits.

#### Distribution.

West Malaysia (Pahang).

### ﻿ Microperus cruralis

Taxon classificationAnimaliaColeopteraCurculionidae

(Schedl, 1975)

1E3BF6A4-9EA7-5BC6-9865-7A53A3E3FCC6

Xyleborus cruralis Schedl, 1975b: 456.
Cnestus cruralis (Schedl): Wood and Bright, 1992: 802.
Coptodryas cruralis (Schedl): Beaver 1995: 201.
Microperus cruralis (Schedl): Smith et al. 2020: 287.
Xyleborus myllus Browne, 1986a: 92. syn. nov.

#### Remarks.

The holotypes of *Xyleborusmyllus* (NHML) and *M.cruralis* (NHMW), and a further specimen in RABC have been compared by RAB, and found to be conspecific. *X.myllus* is accordingly here placed in synonymy with *M.cruralis*.

#### Distribution.

Cambodia, Laos, Thailand.

### ﻿ Microperus exsculptus

Taxon classificationAnimaliaColeopteraCurculionidae

(Eggers, 1927) comb. nov.

904F953D-8966-547E-BB99-7047CCDBDBD4

Figure 6

Xyleborus exsculptus Eggers, 1927: 101.
Coptodryas exsculptus (Eggers, 1927): Wood and Bright 1992: 824.
Xyleborus dentipennis Browne, 1983: 558. syn. nov.

#### Similar species.

*M.armaticeps*, *M.cruralis* (Schedl, 1975).

#### Diagnosis.

2.12 mm long [1.9–2.0 mm Browne (1983)], 2.4× as long as wide. Robust and globular in appearance. This species is distinguished by its large size, stout and robust elytral disc broadly, deeply transversely impressed with a saddle-like depression from scutellum to declivital base; elytral declivity deeply sulcate, its margins begin at interstriae 2 and margined by large tubercles on interstriae 2–6, tubercles on interstriae 1 absent; declivity dull, punctures on declivity small and indistinct; elytral bases slightly emarginated from sutural margin to interstriae 4 to accommodate mycangial tuft, mycangial tuft setae long, very dense; and posterolateral costa absent.

This species is distinguished from *M.cruralis* by its smaller size, elytral bases slightly emarginated from sutural margin to interstriae 4 to accommodate mycangial tuft, elytral disc more deeply depressed, deeper posteriorly, declivital summit without a pair of large tubercles on interstriae 1, margins of declivital sulcus begin at interstriae 2, tubercles on declivital margin slender and acute, and punctures on declivity small and indistinct.

**Figure 6.**
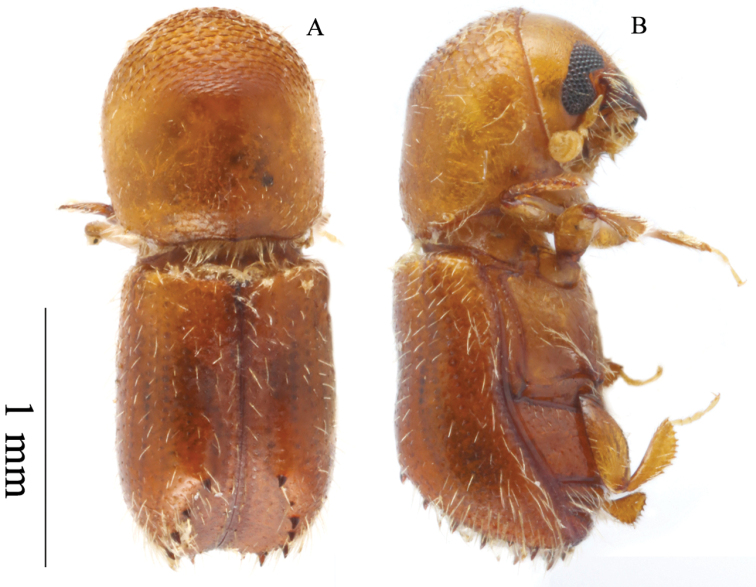
*Microperusexsculptus* (Eggers, 1927) comb. nov. **A** dorsal view **B** lateral view.

#### New records.

Brunei: Kuala Belalong FSC, 116.7°E, 4.34°N, 270 m alt, dipterocarp forest, aerial FIT 4, 17.vi.91, N. Mawdsley (1) (RABC). Thailand: Narathiwat Province, Hala-Bala Wildlife Sanctuary, 5°47'44"N, 101°50'07"E, lowland tropical rainforest, ethanol baited trap, 01.viii.2015, (1) (W. Sittichaya) (WSTC).

#### Distribution.

Brunei, East Malaysia (Sarawak), Philippines, Thailand.

#### Host plants.

This species was collected from a seraya log (*Shorea* sp.) (Dipterocarpaceae) imported to Japan (Browne 1983).

#### Remarks.

The holotype of *Xyleborusdentipennis* (NHML) and images of the *M.exsculptus* lectotype (NMNH) have been examined and compared by RAB and determined to be conspecific. Accordingly, *X.dentipennis* is here placed in synonymy. The species is transferred to *Microperus* based on the characters given for *M.armaticeps*.

### ﻿ Microperus pedellus

Taxon classificationAnimaliaColeopteraCurculionidae

(Schedl, 1969) comb. nov.

3B8A31C4-8752-5EC0-86A2-A0AACAB68F70

Xyleborus pedellus Schedl, 1969: 231.
Coptodryas pedellus (Schedl, 1969): Wood and Bright 1992: 826.

#### Remarks.

The paratype deposited in NHMW was examined by the senior author. This species is included in *Microperus* with the following combination of characters: antennal club truncate, type 2, segment 2 visible on posterior side, dense tuft of setae present along elytral base associated with an elytral mycangium; elytral bases broadly V-shaped, costate; scutellum minute, convex and slightly raised above elytra; pronotum from dorsal view basic and parallel sided (type 2), from lateral view disc longer than anterior slope (type 7), and pronotal base weakly sinuate.

#### Distribution.

Philippines.

### ﻿ Microperus spicatulus

Taxon classificationAnimaliaColeopteraCurculionidae

(Browne, 1986), comb. nov., stat. res.

EE3C67FE-058C-5845-81FE-A96DFA129E02

Xyleborus spicatulus Browne, 1986b: 667.

#### Remarks.

This species was previously considered a synonym of *Coptodryasdentipennis* (= *M.exsculptus*) by Bright and Skidmore (1997: 165). The holotypes of *Xyleborusspicatulus* and *X.dentipennis* (NHML) and further specimens in NHML and RAB, and images of the *M.exsculptus* lectotype (NMNH), have been compared by RAB, and *X.spicatulus* was found to be distinct. It is here recognized as a distinct species and transferred to *Microperus* based on the generic characters given for *M.armaticeps. Microperusspicatulus* can be distinguished from *M.exsculptus* by the following combination of characters (*M.spicatulus* given first): body elongate 3.0–3.1× as long as wide, vs. 2.1–2.2×; larger size (2.2–2.4 mm vs. 1.85–2.0 mm); elytral disc without a discal depression vs. with a discal depression; the number of teeth on each declivital margin 3–5 vs. 7–8.

#### New records.

[Indonesia]: Sumatra, ex *Dipterocarpuss*. [no further data] [1 specimen in NHML determined by F. G. Browne as *Xyleborusdentipennis* Browne, but not that species.] Laos: Sekong prov. ca. 12 km S Seking, Tao Faek waterfalls, 15°14.7'N, 106°45.1'E, 118 m, at light, 8–12.v.2010, J. Hájek (1) (RABC).

#### Distribution.

Indonesia (Sumatra), Laos, East Malaysia (Sabah).

##### Newly recorded species for Thailand

### ﻿ Microperus chrysophylli

Taxon classificationAnimaliaColeopteraCurculionidae

(Eggers, 1930)

6E6F5B52-9299-5C39-8EF8-2FDC2315E026

Figure 7

Xyleborus chrysophylli Eggers, 1930: 205.
Coptodryas chrysophylli (Eggers): Wood and Bright 1992: 823.
Microperus chrysophylli (Eggers): Saha and Maiti 1996: 824.

#### Diagnosis.

2.52–2.6 mm long (2.56 mm; n = 2) 2.83–2.86× as long as wide [(2.6–2.7 mm long (mean = 2.68 mm; n = 5); 2.6–2.7× as long as wide (Smith et al. 2020)]; robust elongate in appearance. This species is distinguished by the elytral disc flat; declivity long, gradual; large size; declivital interstriae 2 lacking granules on declivital face; declivital face strongly sha­greened, weakly impressed along striae 2 and interstriae 2; declivital strial punctures small, indistinct; posterolateral costa granulate; interstriae densely covered with long erect hair-like setae, setae longer than two interstrial widths; and striae setose, setae short, semi-recumbent, as long as strial width.

This species strongly resembles *M.corporaali* and is distinguished by the less strongly sulcate declivity, and declivital strial punctures very large and distinct.

**Figure 7.**
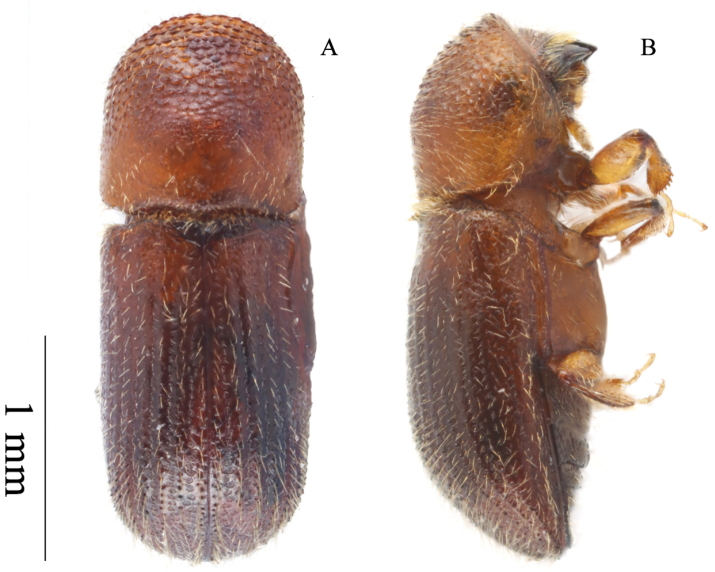
*Microperuschrysophylli* (Eggers, 1930) **A** dorsal view **B** lateral view.

#### New record.

Thailand: Chiang Mai Province, Doi Inthanon National Park, Chomthong District, 18°31'39"N, 98°30'00"E, 1650 msl, Hill evergreen forest, ethanol-baited trap, 01.v.2019 (2) (W. Sittichaya) (WSTC).

#### Distribution.

Bangladesh, China (Yunnan), India (West Bengal) (Smith et al. 2020).

#### Host plants.

This species is recorded from *Cinnamomum* (Lauraceae), *Chrysophyllum* (Sapotaceae), (Maiti and Saha 2004), and *Heveabrasiliensis* (Euphorbiaceae) (Smith et al. 2020).

### ﻿ Microperus nanus

Taxon classificationAnimaliaColeopteraCurculionidae

(Browne, 1949)

6EFACCC7-C528-5A65-9E00-CB969E4A3A4E

Figure 8

Cryptoxyleborus nanus Browne, 1949: 903.
Xyleborus caelator Browne, 1955: 354 (unnecessary replacement name).
Microperus nanus (Browne): Beaver and Hulcr 2008: 151.

#### Diagnosis.

1.12–1.24 mm (mean = 1.19 mm; n = 6), 2.8–3.0× as long as wide; minute species. This species is distinguished by antennal club type 2, segment 2 visible on anterior side, elytra tapering, declivity obliquely attenuate without posterolateral carina, and elytral posterior margin acute.

**Figure 8.**
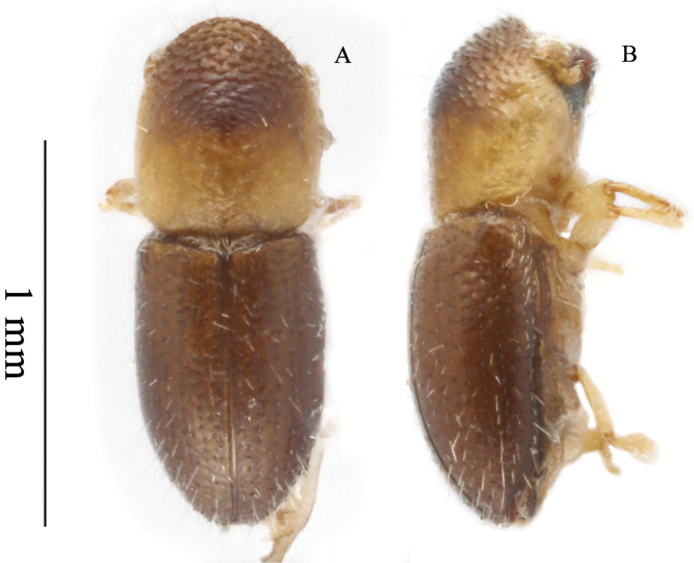
*Microperusnanus* (Browne, 1949) **A** dorsal view **B** lateral view.

#### New record.

Thailand: Narathiwat Province, Hala-Bala Wildlife Sanctuary, 5°47'44"N, 101°50'07"E, lowland tropical rainforest, ethanol-baited trap, 01.xii.2014 (3), 01.iii.2015 (2), (W. Sittichaya) (WSTC).

#### Distribution.

Brunei Darussalam, East Malaysia (Sabah, Sarawak), West Malaysia.

#### Host plants.

This species is only known from Dipterocarp hosts including *Shorea* sp., *Hopeaferrea*, and *Parashoreamalaanonan* (Browne 1961; Beaver and Browne 1979). It is unusual in breeding between the bark and wood, and not making galleries in the xylem (Browne 1961).

### ﻿ Microperus quercicola

Taxon classificationAnimaliaColeopteraCurculionidae

(Eggers, 1926)

CC0E22E6-0EF2-5DE6-AC1E-F000517799F4

Figure 9

Xyleborus quercicola Eggers, 1926: 146.
Microperus quercicola (Eggers): Smith et al. 2018: 396.
Xyleborus izuensis Murayama, 1952: 16. Synonymy: Smith et al. 2018: 396.

#### Diagnosis.

1.8–1.92 (mean = 1.85 mm; n = 4) 2.50–2.53× as long as wide [1.8–2.0 mm long (mean = 1.96 mm; n = 5); 2.38–2.86× as long as wide (Smith et al. 2020)]. This species is distinguished by the elytral disc flat; declivity short, steep; declivity granulate from base to apex, granules small, as abundant as strial punctures; granules dense, separated by the width of one granule; declivital surface shiny; posterolateral costa strongly carinate; interstriae densely setose, setae fine, hair-like as long as the width of an interstria; and strial punctures setose, setae recumbent, hair-like, less than a strial width.

**Figure 9.**
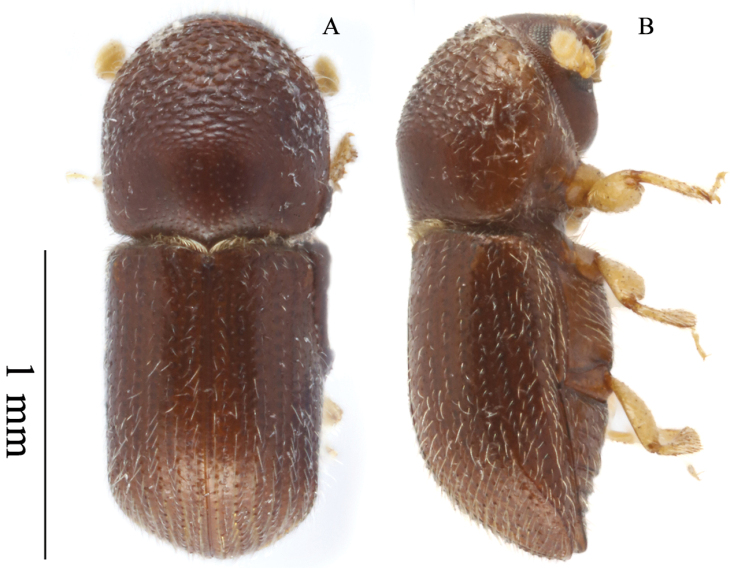
*Microperusquercicola* (Eggers, 1926) **A** dorsal view **B** lateral view.

#### New records.

Thailand: Narathiwat Province, Hala-Bala Wildlife Sanctuary, 5°47'44"N, 101°50'07"E, lowland tropical rainforest, 01.xii.2017 (1 WSTC) ethanol-baited trap; Songkhla Province, Ton Nga Chang Wildlife Sanctuary, tropical rainforest, 6°59'32.1"N, 100°08'57.8"E, 01.ii.2015 (2), ethanol-baited trap, (all W. Sittichaya) (WSTC).

#### Distribution.

China (Guizhou, Hong Kong, Jiangxi, Sichuan, Zhejiang), Japan, Russia (Far East), South Korea, Taiwan.

#### Host plants.

This species is polyphagous and has been recorded from *Cinnamomum* (Lauraceae) (Murayama 1952), *Diospyros* (Ebenaceae), *Fraxinus* (Oleaceae), *Carpinus* (Betulaceae) (Mandelshtam et al. 2018) and “oak trees” (Fagaceae) (Eggers 1926).

## ﻿Discussion

### ﻿Key to *Microperus* species of Indochinese Peninsula and China including species found in Thailand (females only) [modified from Smith et al. (2020)]

**Table:** 

1	Elytral disc broadly, deeply transversely impressed with a saddle-like depression from scutellum to declivital base; declivity deeply sulcate, its margins costate; elytral bases slightly emarginated to accommodate a prominent and dense mycangial tuft	**2**
–	Elytral disc either medially impressed and appearing humped, or flat, or broadly convex; declivity round or flat or convex; elytral bases not emarginated and mycangial tuft minute, either not readily apparent or lightly setose	**3**
2	Elytral base emargination broadly recurved; elytral disc shallowly depressed; declivital summit armed with a pair of large tubercles at interstriae 1; punctures on declivity distinct and broad; larger species, 3.0–3.1 mm	***cruralis***
–	Elytral base emargination broadly V-shaped; elytral disc deeply depressed, more deeper posteriorly; interstriae 1 on declivital summit without a pair of large tubercles; punctures on declivital face small and indistinct; smaller species, 2.12 mm	***exsculptus***
3	Posterolateral margin of elytra smoothly rounded, without any indication of a costa or carina	**4**
–	Posterolateral margin of elytra marked by an elevated costa or carina	**5**
4	Elytral lateral margin tapering posteriorly from the middle to apex; elytral apex acute; minute species, 1.12–1.24 mm	***nanus***
–	Elytral lateral margin subparallel, broadly tapering posteriorly; apex broadly rounded, not acute; larger species, 1.60 mm	***globodeclivis* sp. nov.**
5	Declivity obliquely truncate; posterolateral declivital margin rounded and denticulate	**6**
–	Declivity rounded; posterolateral declivital margin costate or carinate, with or without granules	**9**
6	Declivital interstriae 2 and 3 strongly laterally broadened from base to de­clivital midpoint and then narrowing towards apex	***latesalebrinus***
–	Declivital interstriae parallel from base to apex, never laterally broadened	**7**
7	Denticles on declivital summit and margins larger and more sharply acute than those on declivital face	***kirishimanus***
–	Denticles on declivital summit of equal size and shape as those on declivital face	**8**
8	Denticles on declivital summit as dense as those on declivital face; declivital face opalescent, subshiny	***nudibrevis***
–	Denticles on declivital summit denser than those on declivital face; declivital face shagreened, dull	***perparvus***
9	Larger, 2.55–2.95 mm	**10**
–	Smaller, 1.2–2.1 mm	**12**
10	Stout, 1.93–2.19× as long as wide; elytral posterolateral margin strongly carinate and unarmed	***fulvulus***
–	Elongate, 2.5–2.9× as long as wide; elytral posterolateral margin costate and granulate	**11**
11	Declivital strial punctures very large, distinct	***chrysophylli***
–	Declivital strial punctures small, indistinct	***corporaali***
12	Declivity with granules, denticles or tubercles distinctly less abundant than strial punctures	**13**
–	Declivity with abundant granules or denticles, at least as abundant as strial punctures	**19**
13	Elytral disc shallowly transversely impressed with a saddle-like impression	**14**
–	Elytral disc without a depression	**15**
14	Discal impression deeper, antero-posteriorly narrower, with steeper anterior and posterior slopes, strial punctures on impression with rounded granules; interstrial spines on disc behind impression stronger and backwardly hooked	***sagmatus***
–	Discal impression shallower, antero-posteriorly broader, with gentler anterior and posterior slopes, strial punctures on impression without granules; interstrial tubercles on disc behind impression moderate with rounded apices pointing dorsally	***undulatus***
15	Declivital tubercles uniformly sized	**16**
–	Declivital tubercles not uniformly sized, 1–2 pairs of larger tubercles on declivital interstriae 3	**17**
16	Posterolateral declivital margin carinate, unarmed	***alpha***
–	Posterolateral declivital margin costate and denticulate	***bucolicus* sp. nov.**
17	Declivity steeply rounded, declivital interstriae 1 bearing 1–2 small tubercles on declivital face; larger, 1.9–2.0 mm	***recidens***
–	Declivity gradually rounded, declivital interstriae 1 unarmed on declivital face; smaller, 1.66–1.76 mm	**18**
18	Declivital interstriae 3 with two pairs of prominent denticles; posterior half of elytral disc rugose with deeply impressed striae and costate interstriae, interstriae bearing strong incurved spines that become more prominent toward declivital summit	***serratus* sp. nov.**
–	Declivital interstriae 3 with one pair of prominent tubercles; posterior half of elytral disc smooth, striae and interstriae flush, interstriae with a few small tubercles at summit	***bidentatus* sp. nov.**
19	Elytral disc convex on basal 1/3, appearing humped in lateral view	**20**
–	Elytral disc flat, never appearing humped	**22**
20	Declivital interstriae densely covered with short semi-erect scales	***kadoyamaensis***
–	Declivital interstriae densely covered with long fine, erect hair-like setae	**21**
21	Antennal club flat, type 3 with two sutures visible on apical 1/3 of posterior face; larger, declivity smooth, shiny; larger, 1.95–2.0 mm and more elongate, 2.79–2.86× as long as wide	***minax***
–	Antennal club obliquely truncate, type 2 with segment 1 almost covering posterior face (Fig. 2); one suture visible on posterior face near apex; declivity shagreened, dull; smaller, 1.8–1.9 mm and less elongate, 2.57–2.71× as long as wide	***nugax***
22	Antennal club flat, type 3 with two sutures visible on apical 1/3 of posterior face (Fig. 3	***diversicolor***
–	Antennal club obliquely truncate, type 2 with segment 1 almost covering posterior face; one suture visible on posterior face near apex	**23**
23	Declivital interstrial granules dispersed, separated by the width of at least three granules; posterolateral margin of declivity weakly carinate and granulate; interstrial vestiture consisting of short semi-erect bristles, shorter in length than the width of an interstria; smaller, 1.2–1.7 mm	***pometianus***
–	Declivital interstrial granules dense, separated by the width of one granule; posterolateral margin of declivity strongly carinate; interstrial vestiture consisting of long semi-erect hair-like setae, longer in length than the width of an interstria (easily abraded); larger, 1.8–2.0 mm	***quercicola***

Our study increased the diversity of *Microperus* to 35 species of which 18 are recorded in Thailand. Since 2010, *Microperus* has received considerable attention with eight species described and 12 species transferred from a diversity of genera including *Xyleborus*, *Coptodryas* and *Cyclorhipidion* (Beaver and Liu 2010; Hulcr and Cognato 2013; Beaver et al. 2014; Smith et al. 2018; Park et al. 2020; Smith et al. 2020). However, the faunal emphasis in these studies has largely focused on the faunas of Papua New Guinea, Indochina and the Palearctic. Three of the four new species described herein, and 14 of the 18 Thai species are recorded from the Southern Region (Table 1) which is characterized by species with affinities to the Indo-Malayan and Malayan faunas (Beaver et al. 2014). Based on our findings the *Microperus* faunas of these regions are in need of considerable revision and are undoubtedly more diverse than currently known.

**Table 1.** Synoptic list of the *Microperus* fauna of Thailand.

Species	First Record	Thai distribution
*Microperusalpha* (Beeson, 1929)	Hutacharern et al. (2007)	C: Nakhon Nayok; N:Chiang Mai, Phetchabun; N-E: Chaiyaphum, Nakhon Ratchasima, Sakhon Nakhon
*Microperusbidentatus* Sittichaya, Smith and Beaver sp. nov.	This publication	S: Songkhla, Trang
*Microperusbucolicus* Sittichaya, Smith and Beaver sp. nov.	This publication	S: Nakhon Sri Thammarat
*Microperuschrysophylli* (Eggers, 1930)	New record	N: Chiangmai
*Microperuscorporaali* (Eggers, 1923)	Sittichaya et al. (2012)	S: Chumphon, Nakhon Sri Thammarat
*Microperusdiversicolor* (Eggers, 1923)	Browne (1980)	S: Nakhon Sri Thammarat
*Microperusexsculptus* (Eggers, 1927)	New record	S: Narathiwat
*Microperusglobodeclivis* Sittichaya, Smith and Beaver sp. nov.	This publication	N: Phayao
*Microperusnanus* (Browne, 1949)	New record	S: Narathawat
*Microperusnudibrevis* (Schedl, 1942)	Beaver et al. (2014)	C: Suphanburi; N: Chiang Mai; N-E: Chaiyaphum; S: Nakhon Sri Thammarat, Surat Thani
*Microperusnugax* (Schedl, 1939)	Smith et al. (2020)	C: Chanthaburi; S: Chumphon, Nakhon Sri Thammarat, Surat Thani
*Microperusperparvus* (Sampson, 1922)	Beaver et al. (2014)	C: Nakhon Nayok; N: Chiang Mai, Mae Hong Son, Nan, Phetchabun, Phitsanulok; N-E: Chaiyaphum, Loei; S: Nakhon Sri Thammarat, Surat Thani
*Microperuspometianus* (Schedl, 1939)	Smith et al. (2020)	N: Chiang Mai
*Microperusquercicola* (Eggers, 1926)	New record	S: Songkhla, Narathiwat
*Microperusrecidens* (Sampson, 1923)	Beaver et al. (2014)	C: Phetchaburi; N: Chiang Mai, Nan; S: Chumphon, Nakhon Sri Thammarat
*Microperussagmatus* Smith, Beaver & Cognato (2020)	Smith et al. (2020)	S: Chumphon, Nakhon Sri Thammarat, Songkhla, Surat Thani
*Microperusserratus* Sittichaya, Smith and Beaver sp. nov.	This publication	S: Songkhla, Narathiwat
*Microperusundulatus* (Sampson, 1919)	Beaver et al. (2014)	C: Prachuab Khiri Khan, Uthaithani; N: Chiang Mai; N-E: Chaiyaphum; S: Chumphon, Nakhon Sri Thammarat, Songkhla, Surat Thani, Trang

## Supplementary Material

XML Treatment for Microperus

XML Treatment for Microperus bidentatus

XML Treatment for Microperus bucolicus

XML Treatment for Microperus globodeclivis

XML Treatment for Microperus serratus

XML Treatment for Microperus armaticeps

XML Treatment for Microperus cruralis

XML Treatment for Microperus exsculptus

XML Treatment for Microperus pedellus

XML Treatment for Microperus spicatulus

XML Treatment for Microperus chrysophylli

XML Treatment for Microperus nanus

XML Treatment for Microperus quercicola
